# *Plasmodium falciparum* neonatal malaria with atypical presentation: a case series from southwestern Ethiopia

**DOI:** 10.1186/s12936-024-04987-y

**Published:** 2024-06-05

**Authors:** Zerubabel Girma Tesso, Tariku Yigremachew Gossaye, Dereje Sileshi Bekana, Molla Asnake Kebede, Fikretsion Degemu Besir, Nikodimos Eshetu Dabe

**Affiliations:** 1 School of Medicine, College of Medicine and Health Sciences, Mizan-Tepi University, Mizan Aman, Ethiopia; 2 Department of Pediatrics and Child Health, School of Medicine, Mizan-Tepi University College of Medicine and Health Sciences, Mizan Aman, Ethiopia; 3 Department of Pediatrics and Child Health, School of Medicine, College of Medicine and Health Sciences, Wolkite University, Wolkite, Ethiopia; 4 Department of Biomedical Science, College Medicine and of Health Sciences, Mizan-Tepi University, Mizan Aman, Ethiopia

**Keywords:** Neonatal malaria, Plasmodium falciparum, Atypical presentations, Afebrile

## Abstract

**Background:**

Neonatal malaria is defined as the detection of asexual stages of *Plasmodium* species in the cord blood within the first 28 days of life. It can be congenital or acquired through mosquito bites or blood transfusions. Neonates are generally considered to be relatively protected due to the multiple innate and acquired physiological protective effects present in neonates. However, in areas where malaria is endemic, the prevalence of malaria in neonates is high. The predominant clinical feature of malaria in neonates is fever. Other clinical manifestations of neonatal malaria include respiratory distress, pallor and anaemia, hepatomegaly, refusal to feed, jaundice and diarrhoea. Atypical presentations without fever can lead to inaccurate diagnosis and contribute to neonatal morbidity and mortality. Neonates from endemic areas with any of the above symptoms should be screened for malaria.

**Case presentation:**

We present a series of three cases of neonatal *Plasmodium falciparum* malaria that presented atypically without febrile episodes and were diagnosed and managed at Mizan-Tepi University Teaching Hospital between July and September 2023. The first patient presented with vomiting, refusal to feed, pallor, severe anaemia, and splenomegaly. The second patient presented with an inconsolable cry, failure to pass feces, abdominal distention, and anaemia. The third patient presented with vomiting and anaemia. All patients received a 7-day course of intravenous artesunate; the first patient also received a blood transfusion. All patients recovered and were discharged.

**Conclusions:**

Partial immunity resulting from repeated malaria infections in endemic regions may result in the transfer of high levels of maternal Immunoglobulin G (IgG) antibodies through the placenta and can produce different atypical clinical presentations. In malaria-endemic areas, neonates presenting with any of the presenting signs and symptoms of malaria, including afebrile presentation, require malaria screening to avoid delays in diagnosis.

## Background

Malaria is a protozoal infectious disease caused by *Plasmodium* species, which is common in tropical and subtropical areas of the world [1]. The sub-Saharan African region is responsible for more than 90% of all global malaria cases and deaths related to malaria. Within this region, children under the age of 5 account for 78.1% of all deaths, making it one of the primary causes of death in this age group [2, 3]. Ethiopia accounts for 2.1% of the total cases and 1.7% of the deaths globally [2]. The estimated prevalence of malaria in under-five children is around 22.03% [4]. Approximately 60% of the Ethiopian population lives in malaria-risky areas [5, 6]. Mizan-Tepi University Teaching Hospital is located in a high malaria endemic area that is characterized by stable transmission [6].

Congenital malaria refers to a form of malaria that is transmitted from a mother to her baby via the placenta during pregnancy. Neonatal malaria cases that are diagnosed within the first week of life are classified as congenital malaria since the incubation period must have commenced while the baby was in utero to become clinically evident this early. Typically, congenital malaria shows its first signs or symptoms between 10 and 30 days after birth. Congenital malaria can be diagnosed in neonates over a week old if there's no chance of postpartum infection. Maternal malaria infection during pregnancy is a risk factor for congenital malaria [7, 8]. In a study conducted in a malaria-endemic area, mothers co-infected with HIV and malaria and having severe immunodeficiency (CD4 cell count < 200 cells/mm^3^) are at increased risk for placental malaria and the babies are at increased risk of congenital malaria [9].

Acquired neonatal malaria refers to malaria acquired via mosquito bites or blood transfusion during the first 28 days of life. Residing in malaria-endemic regions, having recently travelled to these areas, and receiving blood transfusions contaminated with plasmodium can all increase the risk of acquired malaria [7, 8]. Neonates and young infants are relatively protected against malaria due to the protective effects of transplacentally transferred maternal antibodies, breast milk components (e.g., lactoferrin, secretory immunoglobulin A), and low levels of para-aminobenzoic acid), high levels of fetal haemoglobin, and the placental barrier. The global prevalence of congenital malaria was 6.9%, ranging from 0.0% in Colombia to 46.7% in Nigeria. The overall prevalence of neonatal malaria was 12.9% [7, 10, 11].

The predominant clinical feature of malaria in neonates is fever (88–100%). Other manifestations include respiratory distress (20–57%), pallor and anaemia (38% each), hepatomegaly (31–80%), refusal to feed (40–70%), jaundice, and diarrhoea (25% each) [12–15]. The signs and symptoms are similar to those of neonatal sepsis and congenital TORCH (Toxoplasmosis, others (syphilis, hepatitis B), Rubella, Cytomegalovirus, Herpes simplex) infections and may cause misdiagnoses. Furthermore, atypical presentations, particularly those without febrile episodes, may result in the inability to suspect malaria or inaccurate alternative diagnosis, contributing to neonatal death and morbidity rates. Neonates from endemic areas with any one of these presenting symptoms require high-index suspicion of malaria and screening.

In this paper, we describe *Plasmodium falciparum* neonatal malaria with atypical clinical manifestations that were treated successfully in the neonatal intensive care unit of the Mizan-Tepi University Teaching Hospital from July to September 2023. The main objective of these case reports is to create a higher index of suspicion among physicians when it comes to diagnosing neonatal malaria.

## Case presentation

### Patient 1

A 25-day-old female baby presented with complaints of vomiting of ingested matter and refusal to breastfeed for 2 days. The baby was born to a para 2 mother after a full-term pregnancy. The mother had a history of *P. falciparum* malaria in the 9th month of pregnancy, 10 days before delivery, and was treated with artemether/lumefantrine. Otherwise, the baby has no fever, cough, fast breathing, or jaundice. On admission, a physical examination revealed a temperature of 37.3, a pulse rate of 179, and a Respiratory Rate of 40. The baby has pallor, splenomegaly, and a weak suckling reflex. Complete blood count showed haemoglobin level of 4.6 g/dl, haematocrit 10.5%, white blood cell (WBC) count of 10.93 × 10^3^/μL (granulocytes 27.3%, lymphocytes 66%, MID 6.7%), and platelet count 320 × 10^3^/μL. No ABO or Rh incompatibility was detected, the TORCH screen was negative, the maternal Venereal Disease Research Laboratory (VDRL) test was nonreactive, and peripheral smear morphology revealed normal and normochromic red blood cells (RBCs). The blood film showed trophozoite stages of *P. falciparum* with a +  + parasite density, i.e. 11–100 parasites per 100 thick film fields. The neonate was transfused with cross-matched whole blood, and treated with intravenous artesunate 3 mg/kg/dose for 7 days, and recovered fully.

### Patient 2

An 18-day-old female neonate was brought to the neonatal unit with complaints of inconsolable crying, failure to pass feces, and abdominal distention of 3 days. The baby had no vomiting, fever, or feeding refusal. The baby was born to a para 2 mother after a full-term pregnancy and passed meconium with in the first 24 h after delivery and had no prior similar episodes of illness. The mother was receiving antenatal care and had no history of malaria diagnosis or malaria symptoms during pregnancy. Physical examination revealed a temperature of 36.8 ºC, a pulse rate of 150, and a respiratory rate of 38. The patient had a protuberant and soft abdomen, and a digital rectal exam revealed stool on the fingers and no blast sign. Complete blood count showed severe anaemia with haemoglobin 11.4 g/dl, haematocrit 32.1%, WBC count of 9.99 × 10^3^/μL(granulocytes 41.3%, lymphocytes 48.9%, MID 9.8%), and platelet count 546,000 × 10^3^/μL. No ABO or Rh incompatibility was detected, the TORCH screen was negative, the maternal VDRL test was nonreactive, and the peripheral smear morphology showed normocytic and normochromic RBCs. The blood film showed trophozoite stages of *P. falciparum*, with a +  + parasite density, i.e. 11–100 parasites per 100 thick film fields. The patient started artesunate intravenous injections. The patient experienced a reduction in abdominal distention and was able to pass stool on the second day of admission. The baby was discharged after 7 days of treatment.

### Patient 3

A 22-day-old male baby presented with a complaint of vomiting of ingested matter of 4–5 episodes per day of 2 days. The baby was from para 3 mother after a full-term pregnancy. The mother was receiving antenatal care and had no history of malaria diagnosis or symptoms of malaria during pregnancy. Physical examination revealed normal vital signs and negligible systemic evaluation. Complete blood count showed a haemoglobin of 12.2 g/dl, haematocrit of 34.6%, WBC count of 9.96 × 10^3^/μL (Granulocytes 42.2%, lymphocytes 48.7%, MID 9.1%) and platelet count of 64 × 10^3^/μL. No ABO or Rh incompatibility was detected, the TORCH screen was negative, the maternal VDRL test was nonreactive, and the peripheral smear morphology showed normocytic and normochromic RBCs. Blood film showed the trophozoite stages of *P. falciparum,* with a + parasite density, i.e. 1–10 parasites per 100 thick film fields. The neonate was treated with 3 mg/kg/dose intravenous artesunate for a total duration of 7 days, and the discharge improved.

## Discussion

Even though one of the mothers in the case series had a malaria diagnosis during pregnancy, it was challenging to determine whether the malaria infection in the case series was congenital or acquired neonatal malaria. Typically, congenital malaria clinically manifests between the 2nd and 4th weeks of life after birth [15]. However, it is important to note that acquired malaria cannot be ruled out. The typical incubation periods after a mosquito bite are 9–14 days for *Plasmodium falciparum*, 12–17 days for *Plasmodium vivax*, 16–18 days for *Plasmodium ovale*, and 18–40 days for *Plasmodium malariae* [15]. The babies in the case series were older than the minimum incubation period of the aforementioned types of malaria.

The most common clinical feature of malaria in neonates is fever (88–100%) [12–15]. In malaria-endemic areas, the usual recommendation is to conduct routine malaria parasite testing on all neonates with fever. Studies conducted in these areas have shown a malaria positivity rate of 4%-17.46% in neonates with fever [16–18]. In this case series, we included neonates who did not present with fever at presentation and during their entire hospitalization and considered them as having an atypical presentation. Fever is often used as the primary indicator to screen for malaria in many studies. However, it is important to note that in malaria-endemic areas neonates who display manifestations, such as anaemia, pallor, poor feeding, jaundice, and other signs and symptoms of illness, even in the absence of fever, should undergo laboratory investigations to rule out the possibility of malaria. It is important to note that the absence of fever does not necessarily mean that malaria can be ruled out. Respiratory distress, anaemia, poor feeding, and hepatosplenomegaly can be the only clinical manifestations of the disease. Previous reports from both endemic and non-endemic regions have described atypical presentations of malaria without febrile episodes [14, 19–22]. Mothers living in malaria-endemic areas can develop partial immunity to the disease after repeated infections, which can protect them from severe attacks and death.

The transfer of high levels of IgG antibodies (which are acquired from repeated infections) from the mother to the fetus provides temporary immunity that leads to delayed presentation after infection and modifies clinical manifestations [14, 19, 20]. The symptoms observed in the cases presented in this case series include poor feeding, vomiting, respiratory distress, anaemia, jaundice, splenomegaly, inconsolable crying, failure to pass feces, abdominal distention, and thrombocytopenia. These symptoms are often non-specific and can be difficult to distinguish from the other differential diagnoses, which poses a significant challenge for healthcare providers. Obtaining a detailed medical history from the mother, including any history of malaria infection during pregnancy. Residence in or recent travel to malaria-endemic areas may aid in the consideration of neonatal malaria as a differential diagnosis. However, a proper laboratory investigation for all possible differential diagnoses is necessary before reaching to diagnosis.

The clinical features of neonatal malaria can be mistaken for neonatal sepsis and congenital TORCH infections, causing a delay in diagnosis [23]. A study carried out in India revealed that the misdiagnosis rate has been steady at around 17%-18% since 2015. The cause for this has been attributed to the overlapping clinical features of different common illnesses in neonates and low parasitaemia, as per reference [7]. Sepsis in neonates requires evidence of systemic inflammatory response syndrome (SIRS) as a prerequisite for meeting the criteria for sepsis. SIRS requires either (1) an abnormal WBC count (total WBC increased or decreased for age or > 10% immature neutrophils); or (2) an abnormal core temperature (> 38.5 °C or < 36 °C), which are absent in our patients [24]. The improvement observed after treatment with only artesunate reduces the likelihood of alternative differential diagnoses.

A study conducted in a malaria-endemic area revealed that newborns can have malaria along with other infections. The study found that out of the newborns with fever, 2.6% had both malaria and a bacterial infection. The common symptoms observed in the babies were fever (100%), fast breathing (50%), jaundice (25.0%), and poor suckling [16]. The study suggests that malaria infection and bacterial sepsis can occur together and have similar symptoms. Therefore, it is recommended that newborns with a fever undergo testing for malaria parasites as part of their diagnostic workup to help prevent delays in administering the appropriate treatment. There is also a case report of the co-occurrence of congenital malaria with tuberculosis [25].

Diagnosis requires a high index of suspicion, so in malaria-endemic areas or neonates whose mothers have recently travelled to such areas, a neonate presenting with any of the signs and symptoms of malaria even in the absence of fever requires proper malaria screening. In this case series, parasitological diagnosis was performed via examination of Giemsa-stained peripheral blood films via microscopy. The World Health Organization recommends the use of malaria rapid diagnostic tests (RDTs) only in the absence of microscopy and for patients who have received incomplete anti-malarial treatment and for whom blood films can be negative [26]. In previous studies, from Nigeria and Burkina Faso, both *P. falciparum* histidine-rich protein 2 (pfHRP2) and lactate dehydrogenase (LDH)-based RDTs showed poor sensitivity in the diagnosis of congenital or acquired neonatal *P. falciparum* malaria and cross-reactivity with transplacentally passed parasite antigens [27, 28]. The highly sensitive and specific PCR testing remains unavailable in resource-limited setups.

The treatment chosen for these patients was based on the National Guidelines and WHO Guidelines for malaria, which is an artesunate Injection is 3 mg/kg administered intravenously at 0 h, 12 h, and 24 h and thereafter, administered once daily for a total duration of 7 days [26]. Except for artesunate + sulfadoxine-pyrimethamine, other artemisinin derivatives are safe and well tolerated by neonates. Eventhough oral artemisinin-based combination therapy is recommended for children weighing less than 5 kg who have uncomplicated malaria or complicated malaria after they can tolerate oral anti-malarial therapy, in this case series we used a full course of IV artesunate instead. Due to their physiological immaturity, the pharmacokinetic and dynamic profiles of anti-malarial drugs may vary among neonates. Slow gastric emptying, villous formation, and intestinal motor activity are not fully developed until week 20 of life and can affect the enteral absorption of most medications [10, 29]. Additionally, the lack of infant formulations of most anti-malarial drugs often necessitates the division of adult tablets, which can lead to inaccurate dosing.

In this case series, the presenting symptoms were resolved within 2–3 days of treatment with the anti-malarial agents. For neonates with severe anaemia, haemoglobin becomes normal after blood transfusion. Due to a lack of packed RBCs at our facility, whole blood was administered instead. The second and third neonates received iron supplements before they were discharged. During the follow-up examination, which was conducted two weeks and at 1 month after discharge, the spleen was not palpable on physical examination for the first patient, and anaemia was corrected for the last two patients.

## Conclusion

Neonatal malaria is a life-threatening condition that can present atypically without febrile episodes. In areas where malaria is endemic, every effort must be made to avoid delays in malaria diagnosis in neonates, which may result in complications and death. By adding malaria tests to regular examinations for newborns with any symptoms of malaria or neonatal sepsis, including afebrile presentations, we can help prevent cases of neonatal malaria from being missed.

## Data Availability

The datasets used and/or analysed during the current study are available from the corresponding author upon reasonable request.

## Abbreviations

IgA: Immunoglobulin A
IgG: Immunoglobulin G
RBCs: Red blood cells
RDTs: Rapid diagnostic tests
SIRS: Systemic inflammatory response syndrome
TORCH: Toxoplasmosis, Others (syphilis, hepatitis B), Rubella, Cytomegalovirus, Herpes simplex
VDRL: Venereal Disease Research Laboratory
WBC: White blood cell
